# Intra-Amniotic LPS Induced Region-Specific Changes in Presynaptic Bouton Densities in the Ovine Fetal Brain

**DOI:** 10.1155/2015/276029

**Published:** 2015-08-31

**Authors:** Eveline Strackx, Reint K. Jellema, Rebecca Rieke, Ruth Gussenhoven, Johan S. H. Vles, Boris W. Kramer, Antonio W. D. Gavilanes

**Affiliations:** ^1^School for Mental Health and Neuroscience, Faculty of Health, Medicine and Life Sciences, Maastricht University and European Graduate, School of Neuroscience (EURON), Universiteitssingel 50, Room 1.152, 6229 MD Maastricht, Netherlands; ^2^Department of Pediatrics-Neonatology, Maastricht University Medical Center (MUMC), Postbus 5800, 6202 AZ Maastricht, Netherlands; ^3^Institute of Biomedicine, Faculty of Medicine, Catholic University of Guayaquil, Avenue Carlos Julio Arosemena, Km. 1 1/2 Via Daule, Guayaquil, Ecuador

## Abstract

*Rationale*. Chorioamnionitis has been associated with increased risk for fetal brain damage. Although, it is now accepted that synaptic dysfunction might be responsible for functional deficits, synaptic densities/numbers after a fetal inflammatory challenge have not been studied in different regions yet. Therefore, we tested in this study the hypothesis that LPS-induced chorioamnionitis caused profound changes in synaptic densities in different regions of the fetal sheep brain. *Material and Methods*. Chorioamnionitis was induced by a 10 mg intra-amniotic LPS injection at two different exposure intervals. The fetal brain was studied at 125 days of gestation (term = 150 days) either 2 (LPS2D group) or 14 days (LPS14D group) after LPS or saline injection (control group). Synaptophysin immunohistochemistry was used to quantify the presynaptic density in layers 2-3 and 5-6 of the motor cortex, somatosensory cortex, entorhinal cortex, and piriforme cortex, in the nucleus caudatus and putamen and in CA1/2, CA3, and dentate gyrus of the hippocampus. *Results*. There was a significant reduction in presynaptic bouton densities in layers 2-3 and 5-6 of the motor cortex and in layers 2-3 of the entorhinal and the somatosensory cortex, in the nucleus caudate and putamen and the CA1/2 and CA3 of the hippocampus in the LPS2D compared to control animals. Only in the motor cortex and putamen, the presynaptic density was significantly decreased in the LPS14 D compared to the control group. No changes were found in the dentate gyrus of the hippocampus and the piriforme cortex. *Conclusion*. We demonstrated that LPS-induced chorioamnionitis caused a decreased density in presynaptic boutons in different areas in the fetal brain. These synaptic changes seemed to be region-specific, with some regions being more affected than others, and seemed to be transient in some regions.

## 1. Introduction

Chorioamnionitis or inflammation/infection of the fetal membranes has been associated with numerous adverse outcomes, like preterm labor and delivery, premature rupture of the membranes, perinatal mortality, and permanent neurological morbidity [1–4]. Several clinical and experimental studies have demonstrated that an intrauterine infection is most closely related to an increased risk for white matter disease and subsequent cerebral palsy [5–7]. Therefore, cerebral palsy has long been the principal neurological outcome of clinical interest.

However, it is increasingly recognized now that neonates exposed to chorioamnionitis are at risk for a whole spectrum of neurobehavioral and cognitive defects [8, 9]. They do not always develop white matter disease but might suffer from grey matter injury, like neuronal programmed cell death, axonal, or dendritic abnormalities, neuronal disorganization, and so forth. [10–13]. In addition, synaptic ultrastructure and density have been shown to be affected after different adverse developmental conditions, like intrauterine growth retardation, malnutrition, and hypoxia [14–22]. Although, it is now accepted that synaptic dysfunction might be responsible for functional deficits, synaptic densities/numbers after a clinical representative fetal inflammatory challenge have not been studied so far.

We therefore aimed to investigate the effect of intra-amniotic inflammation on synaptic density in different brain structures. To study this, pregnant sheep were injected intra-amniotically with lipopolysaccharide (LPS) [11, 23, 24]. In this model, we previously demonstrated that intra-amniotic LPS resulted in an interval-dependent activation of microglia, active astrogliosis, and apoptotic cell death in the brain [11]. In addition, we showed a decrease in synapses expression in the hippocampus [25]. For the current study, we used again synaptophysin immunohistochemistry as an established marker for the quantification of the synaptic density in different regions of the cerebral cortex, the striatum, and the hippocampus. [26–29]. We hypothesized that intra-amniotic inflammation causes a profound loss in synaptic density in different regions of the brain. Synaptic density was assessed after 2 and 14 days of intra-amniotic LPS exposure to test synaptic plasticity following intra-amniotic inflammation.

## 2. Materials and Methods

### 2.1. Animals and Surgical Procedures

**Figure 1:**
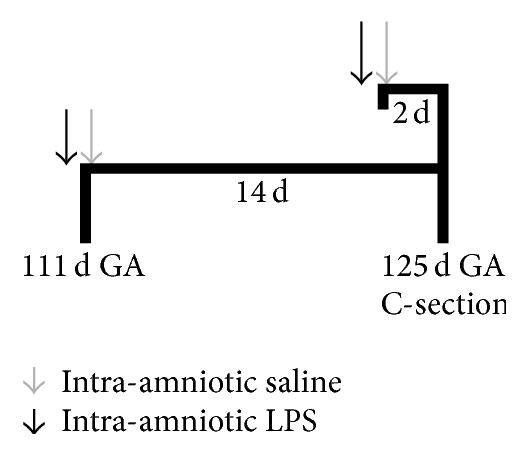
Overview of the experimental setup. Chorioamnionitis was induced by intra-amniotic injections of LPS 2 days (GA = 123 d, *n* = 5) and 14 days (GA = 111 d, *n* = 6) before delivery at GA 125 d (normal GA = 150 d). For the control group (*n* = 7), saline injections were given 2 days or 2 weeks before delivery as well. Endotoxin injections are indicated as black arrows, and saline injections are indicated as grey arrows. GA: gestational age.

All experimental procedures were approved by the Animal Ethics Board of the University of Maastricht on animal welfare according to Dutch governmental regulations. All efforts were taken to minimize the pain and stress levels experienced by the animals and to minimize the number of animals necessary to produce reliable scientific data. Time-mated pregnant Texel ewes bearing both singletons and twins were housed outdoors. Food and water were provided ad libitum. They were randomly assigned to one of the three experimental groups, receiving either LPS or saline. The first group received LPS at day 123 of gestation (*n* = 5), the second group received LPS at day 111 of gestation (*n* = 6), and the third group received a control saline injection at either day 111 or 123 of gestation (*n* = 7) (Figure 1). LPS (10 mg solved in 2 mL sterile and filtered saline) purchased from Sigma (Escherichia coli 055:B5; Sigma Chemical, St. Louis, MO) or saline was injected intra-amniotically under ultrasound guidance [30]. At day 125 of gestation (full-term = 150 days) pregnant ewes were anesthetized and all fetuses were delivered by Caesarean section. Fetuses were killed by a lethal injection of pentobarbital and decapitated. The brain was removed and halved along the midline with one halve being immersion fixed for immunohistochemical analysis. The presence of histological chorioamnionitis was demonstrated by the influx of inflammatory cells into the fetal membranes [31].

### 2.2. Tissue Preparation and Synaptophysin Immunohistochemistry

Brains were embedded in paraffin and cut in 7 *μ*m-thick section using a microtome. Synaptophysin immunostaining was used to analyze presynaptic bouton densities. Synaptophysin is an integral membrane protein located in the synaptic vesicles and an established marker to detect nerve terminals [32]. All sections were processed simultaneously to guarantee identical conditions. First, sections were deparaffinized. All washing and dilutions steps of the antibodies were done by TBS (0.01 M) with 0.2% Triton-X-100 (TBS-T) at room temperature. In order to minimize the background staining, all sections were preincubated with 5% normal goat serum (Sigma, Netherlands) for 30 minutes. Furthermore, normal goat serum (5%) was added to all solutions containing antibodies. An antisynaptophysin antibody (monoclonal mouse; Boehringer-Mannheim, Germany) was used as a primary antibody overnight at a dilution of 1 : 750 at room temperature, followed by immersion with a donkey anti-mouse biotinylated secondary antibody (1 : 100; Jackson laboratories,) for 2 h. Brain sections were further processed using the avidin biotin complex technique with 3,3-diaminobenzidine (DAB) to obtain a color reaction (Vector laboratories, USA). After the labeling procedures, sections were dehydrated in ascending ethanol concentrations, cleared with xylene, and cover-slipped with DePex.

### 2.3. Quantitative Analysis of Synaptophysin Staining

Presynaptic bouton densities were analyzed in different cortical, striatal, and hippocampal areas: (1) motor cortex, (2) somatosensory cortex, (3) entorhinal cortex, (4) piriforme cortex, (5) nucleus caudatus, (6) putamen, (7) CA1-2, (8) CA3, and (9) dentate gyrus. All cortical areas were divided in two: layers 2-3 and layers 5-6. Layers 2 and 3 were taken together because these layers are mainly responsible for corticocortical afferent and efferent connections, while layers 5 and 6 were taken together, because they are the principal source of subcortical efferent connections [33]. Layer 4 was excluded, since, in some areas, like the entorhinal cortex, this layer lacks cell bodies [34].

Identification of the different areas occurred according to the sheep brain atlas of the Michigan State University by anatomical landmarks [35]. For every section (*n* = ±10/animal), measurements were performed for each region of interest in two adjacent areas of 5823 *μ*m^2^. Data of all measurements per subregion of interest per animal were pooled. Data were expressed as numbers of synaptic boutons per square micrometer (1/*μ*m^2^).

The immunoreactive punctae were estimated by calculating the density of the synaptophysin-immunoreactive presynaptic boutons, like previously described by van de Berg et al. [36]. Analysis was done using an Olympus AX-70 microscope. For each subregion, photos were taken from two randomly chosen areas at magnification ×100, using an Olympus F-view cooled CCD camera (Olympus, Tokyo).

The synaptic punctae were detected, using the image analyzing system Cell^P^ (Soft Imaging System, Münster, Germany). All measurements were performed at a single focal plane. Background levels were equalized and a shading correction was carried out by the software to correct for irregularities in illumination. Using a trial and error method, the threshold values, providing the most accurate measurement compared to direct visual counting, were selected. Once the ideal threshold value was found, it was saved in the computer program and kept the same for all measurements. All blood vessels and cell bodies and tissue out of focus were excluded.

### 2.4. Statistical Analysis

All data are represented as mean + standard error of means (+ SEM). For each parameter normality was tested using a Kolmogorov-Smirnov test. All data were normally distributed. Differences in bouton density were tested using a one-way analysis of variance (ANOVA). All significant effects were analyzed in more detail using post hoc Bonferroni tests. The accepted level of statistical significance was *P* < 0.05 for all analyses. All calculations were done using the Statistical Package for the Social Sciences (SPSS 15.0 software, Chicago, IL, USA).

## 3. Results

**Figure 2:**
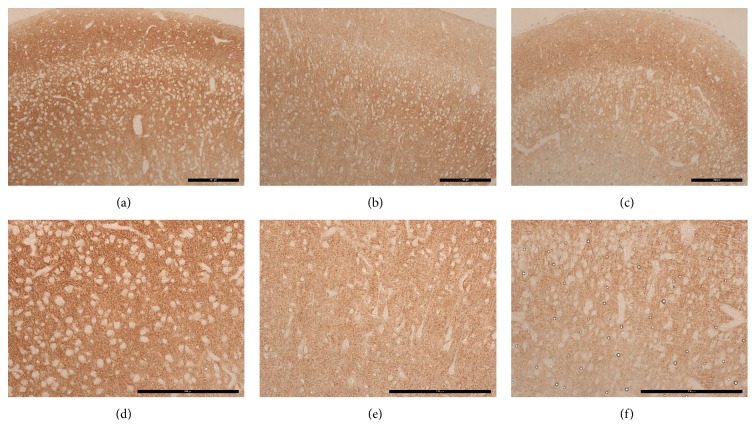
Photographs of synaptophysin immunoreactivity in the motor cortex. (a)–(c) Representative images of synaptophysin immunoreactive presynaptic boutons in the motor cortex (scale bar = 200 *μ*m) of a control (a), a LPS2D animal (b), and a LPS14D animal (c). (d)–(f) Representative images of synaptophysin immunoreactive presynaptic boutons in the motor cortex layers 5-6 (scale bar = 200 *μ*m) of a control (d), a LPS2D animal (e), and a LPS14D animal (f). Intra-amniotic LPS exposure significantly decreased synaptophysin staining in the motor cortex layers 5-6 compared to control animals (see also Figure 3(a)).

**Figure 3:**
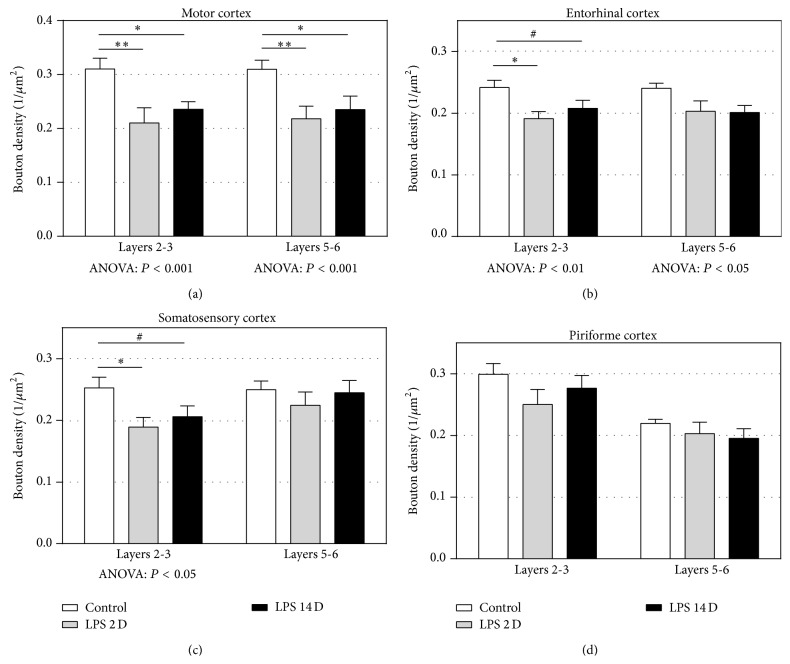
The results of the quantitative analysis of the presynaptic bouton density (1/*μ*m^2^) in layers 2-3 and layers 5-6 of different cortical areas. (a) The mean density of presynaptic boutons in the motor cortex. LPS2D and LPS14D animals showed a significant decrease compared to control animals in both layers. (b) The mean density of presynaptic boutons in the entorhinal cortex. LPS2D animals had a significantly lower density in layers 2-3 compared to controls and LPS14D animals showing a trend. (c) The mean density of presynaptic boutons in the somatosensory cortex. LPS2D animals had a lower density in layers 2-3 compared to controls and LPS14D animals showing a trend. (d) The mean density of presynaptic boutons in the piriforme cortex. All data are expressed as mean + SEM. (^*^ *P* < 0.05; ^**^ *P* < 0.01 and ^#^0.05 < *P* < 0.1).

Figure 2 depicts representative images of synaptophysin immunoreactivity in the motor cortex of control and LPS exposed animals.  Figure 3 shows the results of the quantitative analysis of presynaptic immunoreactivity in the different cortical areas analyzed. The synaptophysin immunoreactivity was restricted to small punctae, representing presynaptic boutons. There were no apparent differences in the morphological appearance of the punctae between groups.

In the motor cortex (Figure 3(a)), the mean presynaptic bouton density significantly decreased in the LPS 2D (*P* < 0.01) and the LPS 14D (*P* < 0.05) group compared to controls in both layers 2-3 and layers 5-6. In the entorhinal cortex (Figure 3(b)) as well as the somatosensory cortex (Figure 3(c)), significantly lower mean presynaptic bouton densities were seen in the LPS 2D group compared to controls in layers 2-3 only (*P* < 0.05). In both cortical areas a trend towards a decreased synaptic density in the LPS14D group was observed as well (0.05 < *P* < 0.1). No significant differences were found in the piriforme cortex (Figure 3(d)).

**Figure 4:**
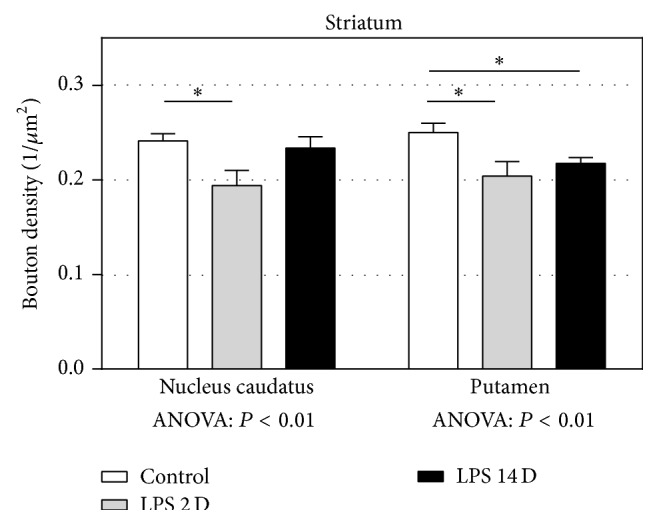
The results of the quantitative analysis of the presynaptic bouton density (1/*μ*m^2^) in the striatum. The mean density of presynaptic boutons in nucleus caudatus and putamen was significantly lower in the LPS2D group compared to controls. In the putamen, there was also a significant decrease in the LPS14D group compared to the control group. All data are expressed as mean + SEM. (^*^ *P* < 0.05).

Figure 4 shows the results for striatum, which was subdivided into the caudate nucleus and the putamen. In both the caudate nucleus and the putamen, there was a significant decrease in the density of presynaptic boutons in the LPS 2D group compared to controls (*P* < 0.05). In the putamen, the mean presynaptic bouton density was significantly lower in the LPS14D group compared to the control group (*P* < 0.05).

**Figure 5:**
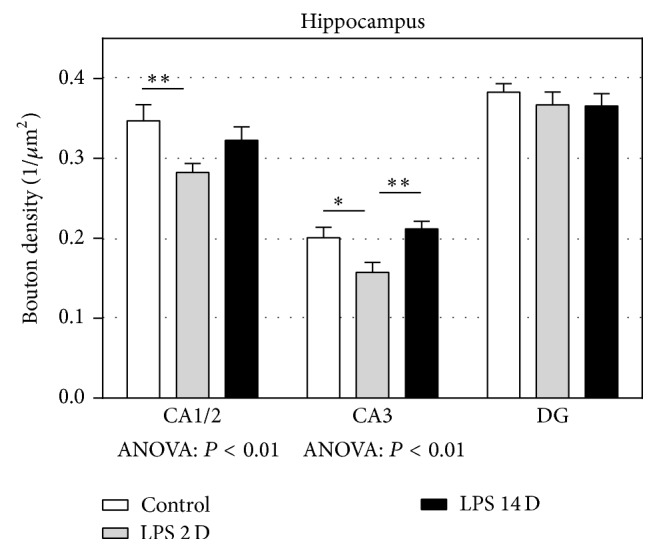
The results of the quantitative analysis of the presynaptic bouton density (1/*μ*m^2^) in the hippocampus. The mean density of presynaptic boutons in CA1/2 area was significantly lower in the LPS2D group compared to controls. In the CA3 area, there was a significant decrease in the LPS2D group compared to both the control and the LPS14D group. No significant differences were found in the DG. All data are expressed as mean + SEM. (^*^ *P* < 0.05; ^**^ *P* < 0.01) CA: cornu ammonis; DG: dentate gyrus.

Figure 5 depicts the results of the presynaptic bouton densities for the different subregions of the hippocampus. In the CA1/2 region, the mean density of presynaptic boutons was significantly less in the LPS 2D group compared to the control group (*P* < 0.01). In the CA3 area, LPS 2D animals had significantly lower bouton densities compared to control animals (*P* < 0.05). In addition, there was a significant difference between the LPS2D and the LPS14D groups, showing that after 14 days the densities were back to control levels (*P* < 0.01). In the dentate gyrus, no significant differences were found between the groups.

## 4. Discussion

The purpose of this study was to assess the effect of intra-amniotic inflammation on synaptic densities in different areas of the sheep brain. Our findings show that LPS-induced intra-amniotic inflammation significantly reduced presynaptic density 2 days after LPS exposure compared to controls in all layers of the motor cortex, in layers 2-3 of the entorhinal and somatosensory cortex, in the CA1/2 and CA3 regions of the hippocampus and the caudate nucleus and putamen. After 14 days of intra-amniotic LPS exposure synaptic density was not significantly lower compared to controls in most regions analysed. This might indicate that the preterm brain is capable of restoring neuronal connectivity after an inflammatory challenge.

Synaptic densities were calculated using an immunohistochemical staining for synaptophysin. This glycosylated protein, located primarily in type I and type II small synaptic vesicles, is an established marker for presynaptic nerve endings. It is important to mention that synaptophysin is present in at least 95% of all neocortical synapses and that its expression profile parallels the time course of synaptogenesis [26–29].

### 4.1. LPS-Induced Chorioamnionitis Caused a Significant Loss in Presynaptic Densities in Different Regions of the Brain

In this study, chorioamnionitis significantly reduced the presynaptic 2 days after LPS exposure compared to controls in all layers of the motor cortex, in layers 2-3 of the entorhinal and somatosensory cortex, and in the CA1/2 and CA3 regions of the hippocampus and the caudate nucleus and putamen. Recently, Soumiya et al. showed that a maternal injection with poly I:C in mice also caused a significant decrease in synaptophysin-positive puncta surrounding cortical neuronal cell bodies [37]. Furthermore no other reports are available on synaptic densities and numbers after a clinical relevant fetal inflammatory challenge. Nevertheless, similar results were found after different adverse developmental conditions, like malnutrition, hypoxia, hypothyroidism, intrauterine growth retardation, and antenatal glucocorticoids [14–21]. For example, Colberg and colleagues found the loss of synaptic densities in layer 3 of the cortex and CA1 of the hippocampus and the caudate nucleus [17, 19] after antenatal betamethasone treatment in sheep. Likewise, lower synaptic densities were found in the visual cortex in growth-retarded fetuses and the hippocampus and cerebellum in neonatal rats after different hypoxic-ischemic insults [18, 20, 21].

The mechanism by which synapses are lost is not known yet. However, in this chorioamnionitis model, the loss of synapses may be regulated by the microglia in the brain. Microglia have shown to make direct contacts with neuronal synapses and after transient cerebral ischemia the duration of these contacts are prolonged and followed by the disappearance of the presynaptic bouton [38]. Therefore activated microglia may be involved in synapse stripping [39]. In addition, synapse loss can be caused by secretion of cytokines as part of the immune response of activated microglia during critical periods of brain and synapse development. In particular, IL1*β* and TNF*α* have been shown to be neurotoxic [40–42]. For example, both TNF*α* and IL1*β* may potentiate glutamate-mediated excitotoxicity and neurodegeneration by increasing NMDA receptor functioning [43–46]. We previously demonstrated in these same animals an activation of microglia in different regions of the brain, like hippocampus, which is consistent with prior work in similar and other animal models [11, 25, 47, 48]. Although we did not directly prove that the microglia are mechanistically involved, these current results point to an activation of this inflammatory cascade in the fetal brain at the moment the synapse loss is present [25]. Whether this inflammatory pathway only is sufficient to induce these synaptic changes or whether this pathway only represents a secondary mechanism awaits further research.

In addition, this reduced presynaptic density after LPS-induced chorioamnionitis in this current study can be the result of several processes. First, different neuronal changes can cause the loss of synapses. For example, degeneration of dendritic structures, loss of dendritic spines, or retraction of axonal processes might cause synapse loss without losing the actual neuron itself [49]. Burd et al. already showed that intrauterine infusion of LPS can cause a significant reduction in the number of dendrites in mice cortical neurons [13]. Furthermore, Kondo and colleagues reported long-term changes in dendritic spines after an intraperitoneal LPS injection in adult mice [50]. Second, lower synaptic densities can be caused by the actual loss of neurons in that specific area as well as by the loss of neurons in regions that have extensive projections to that area. In a previous study, we already demonstrated neuron loss in these animals in the frontal cortex and the hippocampus [11]. Moreover, a prenatal exposure to LPS in rats leads to the loss of striatal dopaminergic neurons [51]. Furthermore, we have to keep in mind that lower synaptophysin-reactivity can also be the result of conformational changes of the protein, alterations in the synthesis, or degradation or a decrease in the amount of presynaptic vesicles instead of only the loss of actual presynaptic terminals [17].

### 4.2. The Reduced Presynaptic Densities Seem to be Region-Specific and Transient in Some Regions

After 14 days of intra-amniotic LPS exposure synaptic density was not significantly lower compared to controls in most regions analysed. This indicates that, after a first injurious hit, the density of presynaptic boutons is restored probably due to the plasticity of the preterm brain. In some region, like the motor cortex and the putamen, however, there is still a significant reduction in presynaptic density in the LPS14D group compared to control animals. Most likely, this region-specific recovery may be due to the maturity of the cerebral area. Cortical neurogenesis occurs later than, for example, hippocampal or striatal neurogenesis [52]. Furthermore, Rees et al. found that the formation of new neuronal processes is retarded if the neurons are particularly vulnerable at the time of the insult but will recover, after a certain delay, if the axonal and dendritic outgrowth is already established and the development is well advanced at that time [53]. The same may hold true for the differences seen in the cortical layers. Cortical layers 2-3 seem to be more affected than cortical layers 5-6. These layers may be more affected, because, at least in humans, synaptogenesis occurs later in the lower layers [54]. In addition, Soumiya et al. also showed that the synaptic development of the upper-layer neurons of the somatosensory cortex seems to be more affected than the deeper-layer neurons in offspring after a maternal viral challenge [37]. In addition, our study shows that the piriforme cortex and the dentate gyrus of the hippocampus seem to be less vulnerable for synaptic alterations at this time point.

### 4.3. Implications and General Conclusion

Recent studies demonstrate that an intrauterine infection associated with preterm birth is related to motor disabilities, as well as learning, social, and other behavioural problems [4, 55–58]. However, not all of these neonates show explicit evidence of structural brain damage, like WMD or periventricular leukomalacia, during ultrasounds or MRI imaging [59, 60]. Therefore, neuronal and grey matter damage may have a significant contribution to these bad outcomes. This presumes that more subtle neuronal injury might be present, like minor neuronal cell loss or synaptic disturbances, which is not always visible during radiological imaging. In this study, we reported the loss of presynaptic terminals in different cortical areas, as well as the hippocampus and striatum. The loss of these presynaptic nerve terminals would suggest functional disturbances in the neuronal network and synaptic transmission. Therefore our results could suggest that this LPS-induced fetal synaptic damage may contribute to the altered neurobehavioural state often described in children exposed to antenatal inflammation by modulating neuronal connectivity.

In conclusion, the present study demonstrated that 2-day intra-amniotic LPS exposure resulted in decreased density in presynaptic boutons in the motor, entorhinal, and somatosensory cortex, in the CA1-2 and CA3 areas of the hippocampus and the nucleus caudate and putamen. These synaptic changes seemed to be region-specific, with some regions being more affected than others, After 14 days of intra-amniotic LPS exposure synaptic density was not significantly lower compared to controls in most regions analysed. This might indicate that the preterm brain is capable of restoring neuronal connectivity after an inflammatory challenge.

Even though these synaptic changes appeared to be transient in some regions, the effects of chorioamnionitis of fetal brain development cannot be disregarded and further research is needed, since these observations could contribute to our understanding of the pathogenesis of the neurobehavioral problems caused by a fetal inflammatory challenge.
